# Genome mining strategies to unlock the metabolic potential of *Streptomyces* sp. VITGV100 (MCC 4961) for next-generation antimicrobial discovery

**DOI:** 10.3389/fmicb.2026.1713836

**Published:** 2026-05-14

**Authors:** Veilumuthu Pattapulavar, Saranyadevi Subburaj, Sathiyabama Ramanujam, Riyanka Shil, Priyanka Velmurugan, Tapas Ghatak, Sanjivkumar Muthusamy, Antony V. Samrot, John Godwin Christopher

**Affiliations:** 1 Department of Biomedical Sciences, School of BioSciences and Technology, Vellore Institute of Technology, Vellore, India; 2 Department of Biotechnology (FoE), Karpagam Academy of Higher Education, Coimbatore, India; 3 Department of Science and Humanities, Karpagam Academy of Higher Education, Coimbatore, Tamil Nadu, India; 4 School of Advanced Sciences, Vellore Institute of Technology, Vellore, Tamil Nadu, India; 5 Department of Microbiology, K.R. College of Arts & Science, Kovilpatti, Tamil Nadu, India; 6 Department of Microbiology, Faculty of Medicine, Manipal University College Malaysia, Melaka, Malaysia

**Keywords:** bioactive metabolites, biosynthetic gene clusters (BGCs), drug discovery, genome mining, microbial cell factories, *Streptomyces* sp. VITGV100, synthetic biology

## Abstract

**Background:**

The rising burden of antimicrobial resistance (AMR) necessitates new bioactive molecules that are both sustainable and therapeutically relevant. Endophytic actinomycetes, particularly *Streptomyces*, represent a valuable but underexplored source of secondary metabolites with pharmaceutical potential.

**Methods:**

An endophytic strain, *Streptomyces* sp. VITGV100, was isolated from tomato stems and subjected to genome sequencing and antiSMASH analysis. A total of 35 compounds were predicted and their associated Biosynthetic gene clusters were identified, including non-ribosomal peptide synthetases, polyketide synthases, RiPPs, terpenes, and melanin. *In silico* pharmacokinetics (SwissADME), target prediction (SwissTargetPrediction, DisGeNET), and pathway enrichment (Reactome) were combined with molecular docking and dynamic simulations to prioritize drug-like metabolites. The antibacterial activity of crude extracts was experimentally evaluated against *Escherichia coli* and *Staphylococcus aureus*.

**Results:**

*In silico* profiling revealed 11 compounds with favorable drug-like properties and no Lipinski violations. Target mapping implicated clinically relevant proteins such as carbonic anhydrase 2 (CA2), PARP1, and PPARA, associated with metabolic disorders, neurodegeneration, and cancer. Docking demonstrated strong binding affinities (−7.4 to −9.6 kcal/mol) for melanin, isorenieratene, and albaflavenone, with stable interactions involving Zn^2+^ coordination and hydrophobic contacts. PASS prediction suggested broad antineoplastic activity, complemented by moderate antibacterial potential. *In vitro* assays confirmed inhibitory zones of 15–20 mm against *E. coli* and *S. aureus*, supporting the computational predictions.

**Conclusion:**

This integrative genome-to-function pipeline establishes *Streptomyces* sp. VITGV100 as a sustainable microbial cell factory for next-generation antimicrobials and multifunctional bioactives. The study highlights the value of combining genome mining, cheminformatics, and experimental validation to unlock cryptic biosynthetic pathways. Future work on metabolic engineering and CRISPR-based refactoring could enhance yields, advancing endophyte-derived *Streptomyces* as renewable platforms for antimicrobial and anticancer drug discovery.

## Introduction

The growing global burden of antimicrobial resistance (AMR) represents one of the most pressing health challenges of the 21st century. The World Health Organization (WHO) warns that resistant infections could cause 10 million deaths annually by 2050 if novel therapeutic strategies are not urgently developed (World Health Organization, 2023). Recent reports in *The Lancet* have underscored the alarming pace at which bacterial pathogens, including *Escherichia coli*, *Klebsiella pneumoniae*, and methicillin-resistant *Staphylococcus aureus* (MRSA), are acquiring resistance mechanisms that undermine the efficacy of frontline antibiotics (Murray et al., 2022). Despite decades of progress in medicinal chemistry, the clinical pipeline for new antimicrobials remains thin, particularly for compounds active against multidrug-resistant Gram-negative bacteria (Butler et al., 2023). Conventional screening strategies have been exhausted, resulting in the rediscovery of known scaffolds rather than the discovery of structurally novel bioactive molecules. This “innovation gap” necessitates a paradigm shift toward genome-guided discovery and the systematic exploitation of microbial secondary metabolism (Dinglasan et al., 2025).

Within this context, actinomycetes, particularly members of the genus *Streptomyces*, remain unrivalled as producers of structurally diverse secondary metabolites. More than two-thirds of clinically used antibiotics—including streptomycin, tetracyclines, and macrolides—originate from *Streptomyces* (Bérdy, 2012). Yet, recent genomic surveys have revealed that the true biosynthetic potential of these organisms remains vastly underexploited. Each *Streptomyces* genome encodes 20–50 Biosynthetic Gene Clusters (BGCs), but only a fraction is expressed under standard laboratory conditions (Rutledge and Challis, 2015). The remainder, often termed “silent” or “cryptic” clusters, represent a rich but hidden reservoir of natural product diversity (Atanasov et al., 2021). Advances in genome sequencing, bioinformatics, and synthetic biology now provide unprecedented opportunities to unlock this latent chemical potential (Groff-Vindman et al., 2025). Tools such as antiSMASH and ARTS allow the rapid prediction and prioritization of BGCs, while metabolic engineering, CRISPR-based activation, and heterologous expression platforms are increasingly used to translate genomic predictions into bioactive molecules (Karthik et al., 2022).

Recent research emphasizes that genome mining is not merely a descriptive exercise but a cornerstone of next-generation antimicrobial discovery pipelines. By integrating genome annotation with cheminformatics, structural prediction, and molecular docking, it is possible to identify drug-like metabolites and assess their potential against validated molecular targets before committing to resource-intensive wet-lab assays. This “genome-to-function” strategy aligns with the design–build–test–learn (DBTL) cycle at the heart of synthetic biology (Spannenkrebs et al., 2024). For example, work by Karthik Loganathan and colleagues highlights how computational mining of *Streptomyces* genomes can be coupled with sustainable cell factory engineering to yield novel bioactives (Gomathinayagam et al., 2022; Rajendran et al., 2024). Similarly, multi-omics-guided genome mining approaches have recently been highlighted as pivotal to bridging the antimicrobial innovation gap (Mitcheltree et al., 2021; Cheng et al., 2024).

Endophytic *Streptomyces* represent particularly promising sources of novel metabolites. Living within plant tissues without causing harm, endophytes are exposed to unique ecological pressures that shape their biosynthetic repertoires (Veilumuthu and Godwin, 2022). Tomato-associated *Streptomyces* strains, for instance, produce a range of bioactives including antifungal polyenes and siderophores with potential agricultural and biomedical applications (Veilumuthu et al., 2022). Yet, most endophytic strains remain genomically uncharacterized, and their secondary metabolic potential is untapped (Pattapulavar et al., 2025b). Harnessing these strains is doubly beneficial: not only do they expand the reservoir of natural products, but they also align with sustainability principles by offering renewable microbial cell factories for drug discovery.

This study focuses on *Streptomyces* sp. VITGV100, an endophytic strain isolated from tomato stems in southern India. Using an integrated pipeline of genome sequencing, BGC mining, *in silico* ADME and target prediction, docking simulations, activity spectrum prediction, and experimental antibacterial assays, we sought to systematically chart and validate the antimicrobial potential of this strain. Our approach exemplifies how endophyte-derived *Streptomyces* can be repositioned as microbial cell factories for next-generation antimicrobials, moving beyond serendipitous discovery to rational prioritization.

Despite extensive studies on actinobacterial secondary metabolism, many biosynthetic gene clusters remain silent under standard cultivation conditions. Genome mining, therefore, represents a powerful approach for uncovering the cryptic metabolic potential of endophytic *Streptomyces*. In this context, the present study combines whole-genome sequencing, BGC annotation, cheminformatics, and preliminary antibacterial assays to explore the biosynthetic potential of *Streptomyces* sp. VITGV100. Our goal is to provide a predictive framework for identifying high-priority clusters and guiding subsequent metabolomic validation.

Beyond its scientific novelty, this work resonates strongly with global sustainability agendas. The United Nations Sustainable Development Goals (SDGs) call for urgent action to combat infectious diseases (SDG 3: Good Health and Well-being) and to foster sustainable industrial innovation (SDG 9: Industry, Innovation, and Infrastructure). Developing sustainable microbial platforms for antibiotic discovery directly contributes to these goals by reducing reliance on environmentally intensive chemical synthesis and by equipping health systems with new therapeutic options to tackle AMR. Importantly, this aligns with the scope of the *Frontiers* Research Topic “Metabolic Engineering and Synthetic Biology for Sustainable Microbial Cell Factories,” which emphasizes the need for innovative microbial platforms in the bioeconomy. By situating *Streptomyces* sp. VITGV100 within this broader framework, our study bridges microbial ecology, genome mining, and synthetic biology to contribute solutions for the global AMR crisis.

The present research builds on the legacy of *Streptomyces* as prolific antibiotic producers while embracing modern genome mining and synthetic biology strategies. By applying a comprehensive genome-to-function workflow, we not only reveal the hidden metabolic capacity of an endophytic *Streptomyces* but also establish a template for sustainable antimicrobial discovery. This approach exemplifies the convergence of microbial genomics, computational biology, and metabolic engineering in addressing one of the most urgent public health challenges of our time.

## Methodology

### Isolation and culturing of endophytic *Streptomyces* sp. VITGV100

Healthy 4-week-old tomato plants (*Lycopersicon esculentum*) were collected from an agricultural field in Madurai, India (9.9420° N, 77.9724° E; 37 ± 2 °C, 12 h light/dark photoperiod). Stem segments (3–5 cm above soil) were surface-sterilized sequentially with 70% ethanol (1 min), 90% ethanol (1 min), 0.9% sodium hypochlorite (4 min), 70% ethanol (30 s), and 10% sodium bicarbonate (5 min), followed by triple rinsing with sterile distilled water. The sterilized tissues were aseptically sectioned and streaked onto ISP2 agar (pH 7.2) supplemented with cycloheximide and nystatin (50 μg/mL each) to inhibit fungal growth. Plates were incubated at 30 °C for 15 days under a 12 h light/dark cycle. Previous studies have shown that plant-associated *Streptomyces* can respond to host-like photic cues, and light–dark cycles do not inhibit growth but can influence endophytic behavior, colonization patterns, and secondary metabolite expression (Cao et al., 2004; Khattab et al., 2016; Kim and Kwak, 2023; Pipinos et al., 2025). This controlled photoperiod, therefore ensured physiological consistency with the ecological niche from which *Streptomyces* sp. VITGV100 was isolated.”

ISP2 agar (pH 7.2) was selected as the primary isolation medium because it is widely used for recovering endophytic *Streptomyces* and maintaining stable morphological traits (Cao et al., 2004; Rashad et al., 2015). Although pH and medium composition can influence secondary metabolite production, systematic media or pH optimization was not performed during the isolation stage. Recent studies have shown that modifications of ISP2, such as supplementation with plant extracts or co-culture stimuli, can significantly enhance metabolite output (Promnuan et al., 2020; Rammali et al., 2024). In this study, ISP2 was therefore used strictly for isolation and activation, whereas metabolite extraction and bioactivity assays were conducted using fermentation broth conditions optimized separately.

These additional screening steps ensured that the selected strain (*Streptomyces* sp. VITGV100) was grown under conditions optimal for subsequent biochemical and genomic colonies with characteristic *Streptomyces* morphology were repeatedly sub-cultured to obtain a pure isolate, designated *Streptomyces* sp. VITGV100 (Veilumuthu and Christopher, 2022).

To obtain contaminant-free plant tissues while preserving internal endophytes, a rigorous but time-optimized surface-sterilization protocol was followed. Sequential exposure to 70 and 90% ethanol for short durations (1 min each) was adapted from established protocols for isolating endophytic *Streptomyces* from tomato roots and stems (Cao et al., 2004; Kim and Kwak, 2023). Reviews on endophyte isolation also recommend ethanol concentrations between 70 and 90% as effective sterilants that do not compromise survival of internal microbial communities when exposure time is carefully controlled (Sahu et al., 2022). Before large-scale isolation, the protocol was validated through pilot trials to ensure that endophyte viability was not affected. Imprint tests and final rinse-plate checks confirmed the complete removal of epiphytic microbes, while internal *Streptomyces* colonies remained recoverable from surface-sterilized tissues. This optimization ensured an effective balance between sterility and preservation of endogenous endophytic populations.

### Genomic DNA extraction, sequencing, and assembly

Genomic DNA from *Streptomyces* sp. VITGV100 was isolated using the CTAB–phenol–chloroform extraction method followed by RNase A treatment. DNA integrity was confirmed by 0.8% agarose gel electrophoresis, and purity was assessed using NanoDrop (A260/280 = 1.82; A260/230 = 1.79). A paired-end shotgun library (2 × 150 bp) was prepared using the Illumina TruSeq Nano DNA Library Prep Kit. Approximately 200 ng of genomic DNA was fragmented to ~350 bp using Covaris M220, followed by end repair, A-tailing, and adaptor ligation. Libraries were size-selected using AMPure XP beads and validated on an Agilent 4,200 TapeStation. Sequencing was performed on the Illumina NextSeq 500 platform, generating ~1.62 Gb of raw data (5,427,160 paired reads). Reads were quality-filtered using Trimmomatic v0.38 to remove adaptors, low-quality bases (QV < 20), and ambiguous reads. High-quality reads (>100 nt) were assembled *de novo* using SPAdes v3.13.0, and scaffolding refinement was performed using GFinisher and SSPACE. Gene prediction was performed using Prokka v1.12, followed by functional annotation using DIAMOND, Blast2GO, and KEGG Automatic Annotation Server (KAAS). The assembled genome was deposited in NCBI under SRA accession SRX11598954 (Pattapulavar et al., 2025a).

### Phylogenetic analysis using 16S rRNA gene

Phylogenetic analysis was performed using the 16 s ribosomal RNA sequences (collected from NCBI) using MegaX. Closely related 16S rRNA gene sequences were retrieved from the NCBI database. Multiple sequence alignment was performed using MEGA X software with the ClustalW algorithm. A phylogenetic tree was constructed using the Maximum Likelihood method with 1,000 bootstrap replicates to assess branch support.

### Secondary metabolite biosynthetic gene cluster analysis

The biosynthetic potential of VITGV100 was evaluated using antiSMASH v6.0, which predicted 35 predicted compounds associated BGCs including NRPS, PKS (types I–III), RiPPs, siderophores, terpenes, ectoine, melanin, and indole derivatives. Comparative analysis identified both highly conserved clusters (e.g., coelichelin, candicidin, geosmin, ectoine) and novel/low-similarity clusters (<30% homology). Antibiotic Resistance Target Seeker (ARTS) was used to prioritize clusters associated with self-resistance genes or proximity to known resistance markers, highlighting BGCs of potential antimicrobial relevance (Veilumuthu et al., 2024). AntiSMASH similarity scores were interpreted according to standard MIBiG-based BGC homology criteria. Similarity percentages reflect gene-level comparisons of core biosynthetic enzymes, domain architectures, and overall cluster organization relative to MIBiG reference clusters. BGCs with ≥70% similarity were classified as high-confidence matches to known pathways, those with 30–70% similarity were considered partially homologous or divergent, and BGCs with <30% similarity were designated as *putatively novel*, reflecting limited conservation of biosynthetic domains and tailoring enzymes. Importantly, low-similarity clusters were *not* assigned any specific chemical structure; instead, they were treated as potentially novel biosynthetic loci.

### Compounds screening and target prediction

Representative metabolites predicted from antiSMASH-annotated predicted compounds respective biosynthetic gene clusters were retrieved from PubChem in canonical SMILES format. A total of 35 predicted compounds BGC of *Streptomyces* sp. VITGV100, obtained from antiSMASH 6.0, was examined for post-screening study using the SwissADME serve (Daina et al., 2017). Initially, we retrieved the canonical SMILES (Simplified Molecular Input Line Entry System) of these metabolites from the PubChem repositories (Wang et al., 2009). By using these SMILES as an input, we have screened the predicted compounds based on the oral bioavailability and drug-likeness parameters (Table 1).

**Table 1:** AntiSMASH-predicted biosynthetic gene clusters (BGCs) in *Streptomyces* sp. VITGV100.

**S. No.**	**Region**	**BGC type**	**Most Similar Known Cluster (MIBiG)**	**Similarity (%)**
1	Region 1.1	NRPS	Coelichelin	100
2	Region 1.3	Lanthipeptide (Class III)	SapB	100
3	Region 5.1	Terpene	Hopene	100
4	Region 33.1	Terpene	Geosmin	100
5	Region 37.1	Indole	7-Prenylisatin	100
6	Region 37.2	Terpene	Isorenieratene	100
7	Region 40.1	Ectoine	Ectoine	100
8	Region 72.1	Terpene	Albaflavenone	100
9	Region 10.1	NRPS-like	Streptothricin	95
10	Region 1.2	NRPS, Lanthipeptide (Class I)	Coelibactin	90
11	Region 31.1	T1PKS, NRPS	Candicidin	90
12	Region 11.2	Siderophore	Desferrioxamine B/E	83
13	Region 2.2	NRPS	CDA (Cda1b/Cda2a/Cda3a)	72
14	Region 41.1	PKS-like, T2PKS, Butyrolactone	Fluostatins M–Q	69
15	Region 15.1	T2PKS	Spore Pigment	66
16	Region 11.1	Melanin	Melanin	60
17	Region 12.1	Lanthipeptide (Class III)	Catenulipeptin	60
18	Region 106.1	T1PKS	Griseochelin	53
19	Region 50.1	Terpene	Carotenoid	45
20	Region 32.2	RiPP-like	Informatipeptin	42
21	Region 5.2	T1PKS	Streptovaricin	31
22	Region 35.1	T1PKS	Nystatin A1	31
23	Region 70.1	Indole	5-Isoprenylindole-3-carboxylate glycoside	23
24	Region 49.1	Terpene	Isorenieratene	18
25	Region 16.1	NRPS-like	Alanylclavam	12
26	Region 111.1	T1PKS	Sanglifehrin A	11
27	Region 2.1	Siderophore	Paulomycin	9
28	Region 28.1	PKS-likeLanthipeptid (Class V)	Methylenomycin A	9
29	Region 4.1	T3PKS	Herboxidiene	8
30	Region 3.1	Terpene	Lysolipin I	4
31	Region 86.1	hglE-KS	2′-Chloropentostatin	6
32	Region 32.1	Terpene	Versipelostatin	5
33	Region 14.1	RiPP-like	No close match in MIBiG database	–
34	Region 9.1	Siderophore	No close match in MIBiG database	–
35	Region 114.1	T1PKS	No close match in MIBiG database	–

Similarity percentages are based on antiSMASH comparison against the MIBiG database. “–” indicates biosynthetic gene clusters with no close known homolog, suggesting potential novelty. NRPS - Non-ribosomal peptide synthetase; T1PKS – Type 1 PolyKetide Synthases; T2PKS – Type 2 PolyKetide Synthases; T3PKS – Type 3 PolyKetide Synthases; RiPP – Ribosomally synthesized and post-translationally modified peptides.

Following structural standardization, canonical SMILES were produced, and each cluster was given a distinct identification number (CT01–CT11). Using the default settings, each SMILES was uploaded separately to the SwissTargetPrediction website (organism: *Homo sapiens*) (Gfeller et al., 2014). Predicted targets, target classes, UniProt identities, and probability scores were obtained and exported for every cluster. A high-confidence target collection was created using a probability cutoff of ≥ 0.10 per cluster. Enrichment analyses were carried out to map the results to pertinent pathways and functional categories after the results were aggregated to find recurrent targets across predicted compounds.

### Disease target prediction

The DisGeNET database was used to investigate disease–gene relationships^1^ for the screened predicted compounds associated with the BGCS (Piñero et al., 2015). To obtain curated and literature-derived illness associations, canonical gene or compound-associated targets that were found in earlier investigations were queried against the platform. Gene symbols and UniProt identifiers were used for searches, and high-confidence connections were ranked by filtering the results by disease category and DisGeNET score. Disease names, source databases, evidence ratings, and ontology IDs were extracted for every query. A thorough disease profile for the targets under study was created by combining the obtained datasets, and overlapping or recurring disease connections across several genes were emphasized. SwissTargetPrediction was used with the default *Homo sapiens* setting, which is recommended for assessing early-phase drug-likeness and identifying putative human protein interactions. The tool does not support bacterial proteome-wide predictions; therefore, predicted targets do not represent antimicrobial-specific mechanisms. Bacterial target prediction was not performed due to the absence of experimentally confirmed metabolite structures.

### Pathway validation

Furthermore, the identified targets are corroborated using the Reactome database (Croft et al., 2011). The Reactome database uses a strict multi-step procedure that includes both computational and manual components. The extensive data model of Reactome records molecular-level occurrences with thorough annotations, such as catalysts, cellular compartments, and circumstances unique to a certain tissue or cell type. Every claim made in Reactome is supported by experimental data obtained either directly from human trials or indirectly from model species in cases where conservation of function is supported by high protein similarity. In our study, the most common protein was found to be Carbonic anhydrase 2 (CA 2) deficiency syndrome is an autosomal recessive disorder that produces osteopetrosis. Further, the corresponding pathway IDs were given as an input for pathway validation.

### Docking simulation

The three-dimensional structures of target proteins were retrieved from the Protein Data Bank (PDB). The PDB structures of selected targets like Carbonic anhydrase 2 (CA2, PDB: 1BCD) and antimicrobial proteins (PDB: 1MWT, 4HL2, 5 M18, and 6RKS) were obtained from the RCSB Protein Data Bank^2^ (Berman et al., 2014). Table 2 shows the functions of 4 different antimicrobial proteins chosen in our study. Based on the resolution and structural cruciality of the targets for this study, every PDB structure was chosen. High-resolution structures reduce positional uncertainty of active-site residues, improving the reliability of predicted ligand binding modes. The crystal structures of target proteins were selected based on good structural resolution datasets. In crystallography, the resolution value (expressed in Ångströms, Å) reflects the level of atomic detail observable in the structure. Lower resolution values (typically 1.2–2.5 Å) indicate greater structural accuracy, clearer electron density, and more reliable placement of amino acid side chains. Therefore, only protein structures with resolutions ≤2.5 Å were used in docking to ensure robust and reproducible interaction modeling. Furthermore, the specific targets chosen in this study were validated using molecular docking.

**Table 2:** List of antimicrobial resistance–associated target proteins included in the molecular docking analysis, showing their biological functions, corresponding PDB structures, and clinical relevance in antimicrobial resistance.

Target Protein	Function	PDB ID	Relevance
Penicillin Binding Protein (PBP2a)	Antibiotic target modification	1MWT	MRSA
NDM-1 β-lactamase	Carbapenem resistance	4HL2	Superbug resistance enzyme
β-lactamase (Class A)	Antibiotic degradation enzyme	5 M18	AMR in *M. tuberculosis*
DNA Gyrase (GyrB)	DNA replication enzyme	6RKS	AMR in Gram-negative

The *in silico* docking was performed for the screened compounds against 5 different proteins using the PyRx server (Dallakyan and Olson, 2015). The docking simulation can now be started in PyRx since the ligand and receptor files were converted to PDBQT format (Protein Data Bank, Q = partial charge, T = AutoDock atom type) prior to docking analysis. The entire docking process is streamlined by PyRx’s graphical user interface. The prepared ligands are chosen for docking, and the receptor is loaded into PyRx as the macromolecule. The defined grid box is applied, and the docking run is started using the Vina Wizard option built into PyRx. To explore various conformational states and binding orientations, the ligand is typically permitted to be flexible throughout the docking process, whereas the receptor is typically retained rigid. The underlying docking engine, AutoDock Vina, determines the binding affinity for each pose by exploring the ligand’s conformational space inside the designated grid box using a stochastic gradient optimization approach. Usually, the docking run produces several poses for each ligand, usually nine, each of which is linked to a binding energy value expressed in kcal/mol. The ideal conformation is typically chosen as the one with the highest expected binding affinity, which is shown by the posture with the largest negative binding energy.

### Post-docking study

The output findings are thoroughly examined after the docking simulation is finished. To choose ligands with the best chance of interacting with the receptor, the docking scores, which stand for the anticipated binding energies, are examined. For a thorough comparison, it is standard procedure to export these results in a CSV file. The best-ranked ligand–receptor complexes were saved and visualized using BIOVIA Discovery Studio Visualizer to analyze binding interactions, hydrogen bonds, and hydrophobic contacts (Baroroh et al., 2023). In this process, we examine the compound interactions that stabilize the complex, such as *π*–π stacking, hydrogen bonds, hydrophobic contacts, and other non-covalent interactions. Redocking of a known ligand with an experimentally proven binding mechanism is a standard procedure for further validation. A dependable docking methodology is generally indicated by an RMSD (*Root-Mean-Square Deviation*) value less than 2 Å, which is subsequently used to compare the re-docking findings against the native or crystallographic posture.

### Antimicrobial activity prediction

The PASS prediction method, which generates activity spectra based on the substance’s chemical structure, is then used to assess the screened compounds (Lagunin et al., 2000). Two major probabilities, Pa (*Probability of Activity*) (the likelihood that the substance is active) and Pi (*Probability of Inactivity*) (the likelihood that it is inert), are produced by PASS and range from 0 to 1. Since they are computed separately, these odds typically do not add up to one. According to the interpretation guidelines, there is a high probability of discovering the predicted activity experimentally if P_a_ > 0.7; a moderate probability if P_a_ < 0.7, but the compound is probably structurally distinct from known actives; and a low probability if P_a_ < 0.5, but the compound may also have a novel structure. Crucially, only those activities are deemed meaningful for prediction when P_a_ > P_i_. Numerous biological functions, including antibiotic efficacy, are predicted by the PASS server. Our study has projected activities as a flexible guide for experimental validation, forming an intrinsic property spectrum of the compounds.

### Metabolite production from *Streptomyces* sp. VITGV100

Pure cultures of *Streptomyces* sp. VITGV100 were cultivated in ISP2 broth (200 mL in 1 L Erlenmeyer flasks) at 30 °C for 15 days with continuous agitation at 120 rpm to ensure adequate aeration, after which the biomass was separated by centrifugation (10,000 rpm, 20 min). Secondary metabolites were recovered from the culture supernatant through liquid–liquid extraction with ethyl acetate (1:1, v/v). The organic fraction was shaken for 24 h at 200 rpm, allowed to settle, and subsequently concentrated under reduced pressure in a rotary evaporator at 54 °C.

### Antimicrobial activity of *Streptomyces* sp. VITGV100

The antimicrobial activity of the crude ethyl acetate extract of *Streptomyces* sp. VITGV100 was evaluated using the agar well diffusion assay. The crude extract was re-dissolved in dimethyl sulfoxide (DMSO) to obtain a stock solution of 1 mg/mL. Mueller–Hinton agar plates were seeded with freshly prepared bacterial suspensions (10^6^ CFU/mL) of *Bacillus subtilis* (MTCC 2756), *Staphylococcus aureus* (MTCC 737), *Escherichia coli* (MTCC 1687), and *Pseudomonas aeruginosa* (MTCC 7815). Wells of 6 mm diameter were aseptically punched into the agar, and 25, 50, 75, and 100 μL of the crude extract solution were carefully dispensed into the respective wells. DMSO served as the negative control, while tetracycline was used as the positive control. The plates were incubated at 37 °C for 24 h, after which the zones of inhibition were measured. All assays were performed in triplicate, and the results were expressed as mean ± standard deviation (Veilumuthu and Godwin, 2022; Pattapulavar et al., 2025a; Pattapulavar et al., 2025b).

### GC–MS identification and quantification

Chemical constituents were profiled by GC–MS (Thermo Scientific Trace GC Ultra, ISQ Quadrupole MS) equipped with a TG-5MS capillary column, using helium as the carrier gas (1 mL/min) and electron ionization at 70 eV. The oven program started at 50 °C (2 min hold), ramped to 150 °C at 7 °C/min, then to 270 °C at 5 °C/min, and finally to 310 °C at 3.5 °C/min. Compounds were annotated against the NIST library. GC–MS data processing and compound identification were performed using the NIST 14 Mass Spectral Library. A minimum NIST match factor threshold of ≥ 80% was applied for accepting compound identity, while values ≥ 90% were considered high-confidence identification. Only peaks satisfying these criteria were included in the final metabolite list. Quantification was carried out using relative peak area normalization, where the area of each chromatographic peak was expressed as a percentage of the total ion chromatogram (TIC). No external standard calibration was performed, and results are therefore interpreted as semi-quantitative abundances consistent with standard natural-product GC–MS workflows.

### LC–MS analysis of the extract of *Streptomyces* sp. VITGV100

LC–MS analysis of the crude ethyl acetate extract of *Streptomyces* sp. VITGV100 was performed following a modified protocol of Um et al., (2022). Separation was achieved using a Phenomenex Kinetex C18 column (50 × 2.1 mm, 2.6 μm, 100 Å). Mass spectrometric detection was carried out using a QTOF mass spectrometer equipped with an electrospray ionization (ESI) source operating in negative ion mode. The instrument parameters were set as follows: capillary voltage 4.5 kV, source temperature 200 °C, and scan rate 1 Hz. Data were acquired over a wide mass range to capture low- and high-molecular-weight secondary metabolites. LC–MS/MS fragmentation spectra were collected for major precursor ions to assist in compound class annotation.

## Result

Our workflow integrates genome mining, cheminformatics, molecular docking, metabolite profiling, and antimicrobial assays. Each computational prediction step informed the subsequent experimental validation, enabling a systematic discovery of bioactive metabolites from *Streptomyces* sp. VITGV100.

### Genome features of *Streptomyces* sp. VITGV100

Whole-genome shotgun sequencing of *Streptomyces* sp. VITGV100 generated 1.62Gb of high-quality paired-end reads (5.4 million reads). *De novo* assembly using SPAdes produced 343 scaffolds, with a total genome length of 8.28 Mb, GC content of 73.1%, N50 of 96,723 bp, and a maximum scaffold size of 336,030 bp. Reference-guided finishing with GFinisher and SSPACE further refined the assembly to 129 scaffolds, corresponding to a final draft genome size of 7.96 Mb, with an improved N50 of 110,653 bp and a maximum scaffold length of 443,650 bp. Gene prediction identified 7,045 genes, including 6,895 protein-coding sequences, 5 rRNAs, and 85 tRNAs. Functional annotation showed that ~96% of genes had significant BLAST hits, predominantly matching *Streptomyces* spp. KEGG pathway mapping assigned 2,243 genes across major metabolic and secondary metabolite biosynthesis pathways.

### Phylogenetic analysis using 16S rRNA gene

**Figure 1:**
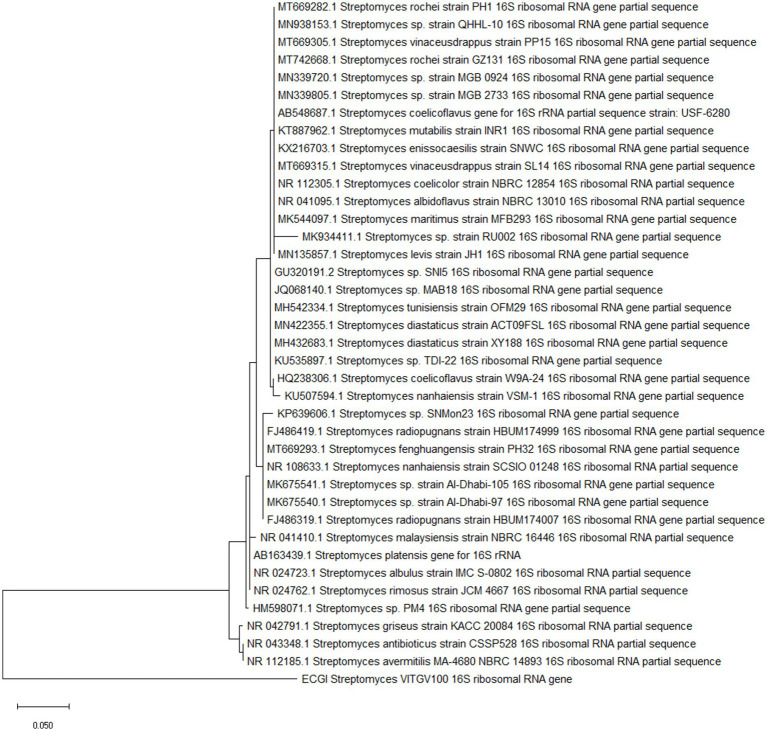
Phylogenetic analysis of 16S ribosomal sequence of *Streptomyces* sp. The tree was constructed using MegaX using maximum likelihood method. *Streptomyces* sp. VTVGV100 is highlighted.

Phylogenetic analysis performed using 16 s ribosomal RNA sequences revealed the distinct evolutionary status of this strain. *Streptomyces* sp. VTVGV100 falls on a separate branch which shows their divergent evolutionary. In phylogenetic tree *Streptomyces avermitilis* and *Streptomyces antibioticus* falls in branches near to *Streptomyces* sp. VTVGV100 which shows they are closely related (Figure 1).

### AntiSMASH analysis of BGC

Importantly, antiSMASH 6.0 analysis identified an exceptionally rich repertoire of 35 predicted compounds associated biosynthetic gene clusters (BGCs), including NRPS, PKS (Types I–III), terpenes, RiPPs, siderophores, ectoine, indole, and melanin pathways. These genomic features highlight the strain’s high potential for producing structurally diverse and pharmaceutically relevant secondary metabolites. The complete list of predicted metabolites and their closest characterized analogs is summarized in Table 1. Of the predicted BGCs, clusters exhibiting <30% similarity to MIBiG entries were classified as *putatively novel*, based on minimal conservation of core enzymatic domains and cluster architecture. These low-similarity BGCs likely represent unexplored or taxonomically restricted pathways, but due to insufficient homology, no specific metabolite identity was inferred.

To prioritize the predicted metabolites for downstream biological relevance, the identified BGC products were subjected to cheminformatics-based target prediction and activity assessment.

### Pharmacokinetic analysis

In SwissADME analysis, we screened a total of 11 predicted compounds, which had zero Lipinski violations, indicating good oral drug-likeness in the early stages (Table 3). Target profiles and disease associations correspond to individual compounds, not to the BGCs themselves. Even though it shows favorable physicochemical properties for absorption still needs to be proven by an *in vitro* study. To confirm their potential for therapeutic development, more analysis of their solubility, permeability, metabolism, and synthetic accessibility is needed. Further, these compounds were taken forward for target identification using the Swiss TargetPrediction tool. For BGCs showing low similarity to known MIBiG entries (<30%), the structures of reference compounds (e.g., Herboxidiene, Methylenomycin A) were not interpreted as the exact metabolites produced by *Streptomyces* sp. VITGV100. Instead, these molecules were used as structural analogues to explore the potential chemical space and biological target classes associated with the corresponding BGC families. This approach is consistent with the similarity-property principle in chemoinformatics, which states that structural analogues— including those with low numerical similarity—may retain comparable physicochemical or functional attributes due to shared substructures or scaffolds (Wermuth, 2006; Weber et al., 2015; López-Pérez et al., 2024). Recent advances in chemical language models further demonstrate that compounds with low structural similarity can display convergent functional activity (Kosonocky et al., 2023).

**Table 3:** Pharmacokinetic analysis of the 11 compounds.

**S. No.**	**Compounds**	**PubChem IDs**	**Canonical SMILES**	**Lipinski #violations**
1.	Herboxidiene	6,438,496	CO[C@H]([C@H]([C@H]1O[C@]1(C)C[C@@H](/C=C/C=C(/[C@H]1O[C@H](CC[C@@H]1C)CC(=O)O)\C)C)C)[C@H](O)C	0
2.	Melanin	6,325,610	Cc1c2[nH]cc3c2c(c(=O)c1 = O)c1c[nH]c2c1c3c(=O)c(=O)c2C	0
3.	Alanylclavam/2	194,618	OC(=O)[C@H](C[C@@H]1O[C@@H]2 N(C1)C(=O)C2)N	0
4.	Methylenomycin A	10,899,285	OC(=O)[C@@H]1C(=C)C(=O)[C@@]2([C@@]1(C)O2)C	0
5.	Geosmin	29,746	C[C@H]1CCC[C@@]2([C@]1(O)CCCC2)C	0
6.	7-Prenylisatin	122,397,888	CC(=CCc1cccc2c1NC(=O)C2 = O)C	0
7.	Isorenieratene	9,984,420	C/C(=C\C=C\C=C(\C=C\C=C(\C=C\c1c(C)ccc(c1C)C)/C)/C)/C=C/C=C(/C=C/c1c(C)ccc(c1C)C)\C	0
8.	Ectoine	126,041	OC(=O)[C@@H]1CCN=C(N1)C	0
9.	Carotenoid	11,227,325	OC/C(=C\C=C\C(=C\C=C\C=C(\C=C\C=C(\C(=O)/C=C/1\C(C)(C)C[C@@H](C[C@@]1(C)O)O)/C)/C)\C)/C#CC1 = C(C)C[C@H](CC1(C)C)O	0
10.	Albaflavenone	25,137,938	O=C1C[C@@H]([C@@]23C1 = C(C)C(C)(C)[C@H](C3)CC2)C	0
11.	2’-Chloropentostatin	341,960	OCC1OC(C(C1O)Cl)n1cnc2c1NC=NCC2O	0

### Target identification

The SwissTargetPrediction analysis produced the 11 predicted compounds that shared several protein targets, which have therapeutic significance. Potential osteopetrosis and diuretic applications are suggested by the majority’s projected interactions with carbonic anhydrases (CA1, CA2, CA9, and CA12), which are enzymes involved in pH regulation and tumor microenvironment adaptation. Apart from that, GABA receptor subunits (GABRB2, GABRG2), PPARA (lipid metabolism and energy regulation), EPHX2 (epoxide metabolism), and PARP1 targets were identified from the 11 predicted compounds (Table 4). SwissTargetPrediction was performed separately for each of the 11 selected compounds. All 11 compounds were analyzed for target identification using SwissTargetPrediction. In this, each compound yielded multiple predicted targets. From these, we have selected only the common targets that were found in more than 5 of the compounds. Docking protocol validation by re-docking native ligands showed that 11 targets re-docked with RMSD ≤ 2.0 Å, supporting the accuracy of the grid definitions and docking parameters (Supplementary Table S1).

**Table 4:** Common target prediction obtained from 11 compounds using the SwissTargetPrediction server.

**Uniprot ID**	**Protein name**	**Gene symbol**
P00918	Carbonic anhydrase 2	CA2
P00915	Carbonic anhydrase 1	CA1
P47870	Gamma-aminobutyric acid receptor subunit beta-2	GABRB2
P18507	Gamma-aminobutyric acid receptor subunit gamma-2	GABRG2
P09917	Polyunsaturated fatty acid 5-lipoxygenase	ALOX5
Q07869	Peroxisome proliferator-activated receptor alpha	PPARA
P34913	Bifunctional epoxide hydrolase 2	EPHX2
P09874	Poly [ADP-ribose] polymerase 1	PARP1
P56817	Beta-secretase 1	BACE1
P0DMS8	Adenosine receptor A3	ADORA3
O43570	Carbonic anhydrase 12	CA12
Q16790	Carbonic anhydrase 9	CA9
P06276	Cholinesterase	BCHE
P11309	Serine/threonine-protein kinase pim-1	PIM1
P18031	Tyrosine-protein phosphatase non-receptor type 1	PTPN1
P27338	Amine oxidase [flavin-containing] B	MAOB

This table presents the merged set of predicted targets obtained from individual SwissTargetPrediction analyses of all 11 compounds. Each entry corresponds to a protein predicted as a potential target across more than 5 compounds.

### DisGeNET

**Figure 2:**
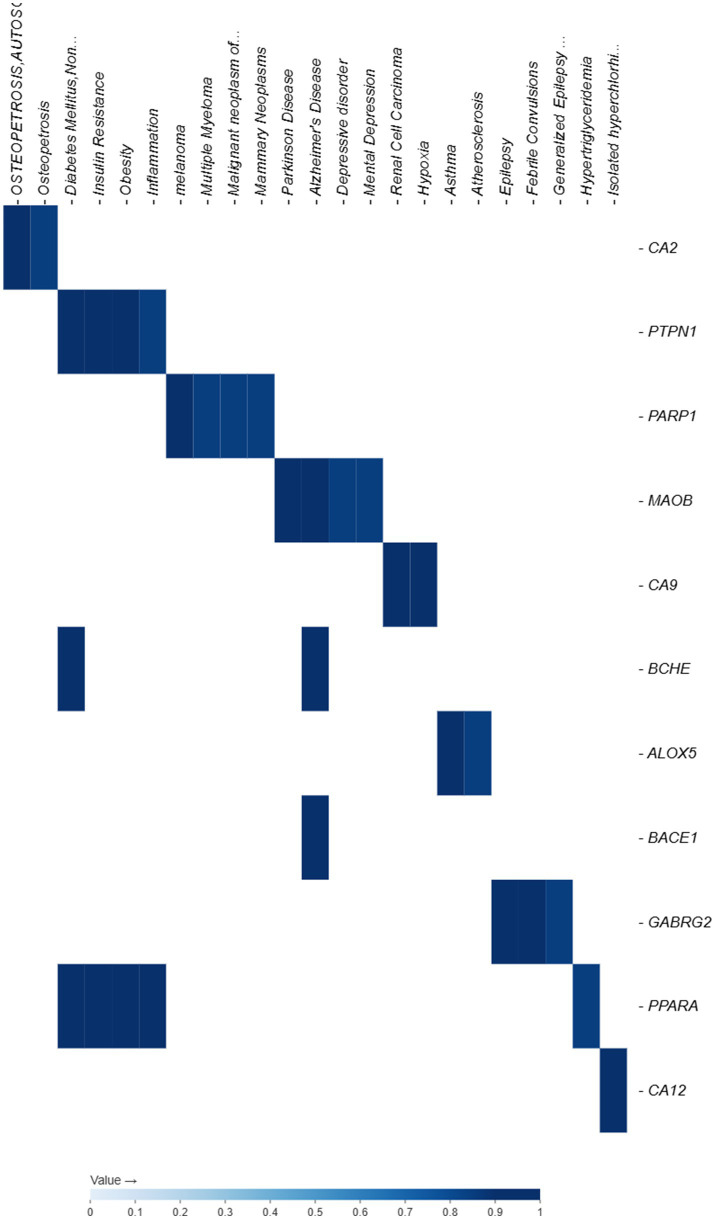
Gene-disease association of the common targets.

From the DisGeNET analysis, we found that all 11 predicted compounds projected targets have high confidence scores (≥0.95) and are highly associated with clinically relevant illnesses (Table 5). The pathogenic significance of CA2 was highlighted by the fact that it was the most frequently found and consistently linked to osteopetrosis, Autosomal Recessive 3 (score 1.0). Major disorders, including melanoma (PARP1), renal cell carcinoma (CA9), Alzheimer’s disease (BACE1 and BCHE), Parkinson’s disease (MAOB), type 2 diabetes mellitus (PTPN1), asthma (ALOX5), and obesity (PPARA), have been associated with other targets. With CA2 emerging as a prominent shared disease-associated target, these relationships highlight the drug’s potential therapeutic significance in a variety of illnesses encompassing metabolic, neurological, inflammatory, and cancer-related diseases. Moreover, the most frequent target, CA2, was consistently linked to Autosomal Recessive 3 Osteopetrosis (score 1.0), highlighting its significance with a pathway ID of R-HSA-1266738, R-HSA-1430728, and R-HSA-382551 (Table 5). These results demonstrate the compound’s wide range of therapeutic promise for conditions linked to metabolism, neurodegeneration, inflammation, and cancer. In our study, we have chosen the most common CA2 gene, which is responsible for osteopetrosis, an autosomal recessive 3 (OPTB3) severe genetic disorder caused by mutations in the CA2 gene. Therefore, in our study, we have chosen CA2 for further analysis. Figure 2 exemplifies the gene-disease association of the common targets.

**Table 5:** Gene disease association for the identified targets and their pathway IDs.

**Gene symbol**	**Disease name**	**Score**	**Pathway Id**
**Ca2**	**Osteopetrosis, Ar 3**	**1.0**	R-Hsa-1266738, R-Hsa-1430728, R-Hsa-382551
**Ca2**	**Osteopetrosis, Ar 3**	**1.0**	R-Hsa-1266738, R-Hsa-1430728, R-Hsa-382551
Parp1	Melanoma	1.0	R-Hsa-162582, R-Hsa-1643685, R-Hsa-392499, R-Hsa-73894, R-Hsa-74160
**Ca2**	**Osteopetrosis, Ar 3**	**1.0**	R-Hsa-1266738, R-Hsa-1430728, R-Hsa-382551
**Ca2**	**Osteopetrosis, Ar 3**	**1.0**	R-Hsa-1266738, R-Hsa-1430728, R-Hsa-382551
Bche	Alzheimer’s Disease	1.0	R-Hsa-112316, R-Hsa-1430728, R-Hsa-392499, R-Hsa-9748784
**Ca2**	**Osteopetrosis, Ar 3**	**1.0**	R-Hsa-1266738, R-Hsa-1430728, R-Hsa-382551
**Ca2**	**Osteopetrosis, Ar 3**	**1.0**	R-Hsa-1266738, R-Hsa-1430728, R-Hsa-382551
Bace1	Alzheimer’s Disease	1.0	R-Hsa-392499
Ca2	Osteopetrosis, Ar 3	1.0	R-Hsa-1266738, R-Hsa-1430728, R-Hsa-382551
Maob	Parkinson Disease	1.0	R-Hsa-1430728
**Ca2**	**Osteopetrosis, Ar 3**	**1.0**	R-Hsa-1266738, R-Hsa-1430728, R-Hsa-382551
**Ca2**	**Osteopetrosis, Ar 3**	**1.0**	R-Hsa-1266738, R-Hsa-1430728, R-Hsa-382551
**Ca2**	**Osteopetrosis, Ar 3**	**1.0**	R-Hsa-1266738, R-Hsa-1430728, R-Hsa-382551
**Ca2**	**Osteopetrosis, Ar 3**	**1.0**	R-Hsa-1266738, R-Hsa-1430728, R-Hsa-382551
**Ca2**	**Osteopetrosis, Ar 3**	**1.0**	R-Hsa-1266738, R-Hsa-1430728, R-Hsa-382551
**Ca2**	**Osteopetrosis, Ar 3**	**1.0**	R-Hsa-1266738, R-Hsa-1430728, R-Hsa-382551
**Ca2**	**Osteopetrosis, Ar 3**	**1.0**	R-Hsa-1266738, R-Hsa-1430728, R-Hsa-382551
Ptpn1	Dm, Nid	1.0	R-Hsa-109582, R-Hsa-162582, R-Hsa-168256, R-Hsa-74160, R-Hsa-8953897
Alox5	Asthma	1.0	R-Hsa-1430728, R-Hsa-168256
**Ca2**	**Osteopetrosis, Ar 3**	**1.0**	R-Hsa-1266738, R-Hsa-1430728, R-Hsa-382551
**Ca2**	**Osteopetrosis, Ar 3**	**1.0**	R-Hsa-1266738, R-Hsa-1430728, R-Hsa-382551
Ca9	Rcc	1.0	R-Hsa-1430728, R-Hsa-8953897
Bace1	Alzheimer’s Disease	1.0	R-Hsa-392499
Bche	Alzheimer’s Disease	1.0	R-Hsa-112316, R-Hsa-1430728, R-Hsa-392499, R-Hsa-9748784
Maob	Parkinson Disease	1.0	R-Hsa-1430728
Ptpn1	Dm, Nid	1.0	R-Hsa-109582, R-Hsa-162582, R-Hsa-168256, R-Hsa-74160, R-Hsa-8953897
Alox5	Asthma	1.0	R-Hsa-1430728, R-Hsa-168256
Ppara	Dm, Nid	0.95	R-Hsa-1266738, R-Hsa-1430728, R-Hsa-1852241, R-Hsa-392499, R-Hsa-74160, R-Hsa-8953897
Ppara	Obesity	0.95	R-Hsa-1266738, R-Hsa-1430728, R-Hsa-1852241, R-Hsa-392499, R-Hsa-74160, R-Hsa-8953897

Bolded genes CA2 were identified as the most common disease with the 11 predicted compounds. AR 3 **-** Autosomal Recessive 3; DM **-** Diabetes Mellitus; NID **-** Non-Insulin-Dependent; RCC **-** Renal Cell Carcinoma.

### Pathway analysis

For pathway validation using the Reactome database, the pathway IDs of the CA2 gene (R-HSA-1266738, R-HSA-1430728, and R-HSA-382551) were used as input. Osteopetrosis is a developmental bone disorder characterized by excessive bone mass and increased fragility resulting from impaired osteoclast function and differentiation. Defective osteoclasts lead to inefficient bone resorption, producing dense but fragile “marble-like” bones. This condition also disrupts normal bone modeling, where the expansion of the marrow cavity is essential for proper bone formation. Failure of bone resorption prevents the development of marrow cavities, affecting bone marrow development and leading to hematopoietic failure in severe infantile cases.

The pathway ID R-HSA-1266738 highlights key developmental pathways in the human embryo, including stem cell regulation, gastrulation, and blastocyst development. These processes involve transcriptional regulation, signaling cascades, and HOX gene activation that guide lineage specification and organogenesis. Together, these pathways regulate the development of essential organ systems such as the kidney, heart, blood, muscle, and skin tissues. From this analysis, osteopetrosis can be understood as a disruption of normal bone formation caused by malfunctioning osteoclasts, which are responsible for bone resorption during skeletal development.

Similarly, the pathway ID R-HSA-1430728 illustrates major metabolic pathways mapped in the Reactome database, including carbohydrate, lipid, amino acid, nucleotide, and vitamin/cofactor metabolism. Osteopetrosis is considered an inherited metabolic disorder associated with abnormalities in skeletal metabolism and hematopoiesis during development. This pathway emphasizes the essential roles of metabolic intermediates such as pyruvate, acetyl-CoA, and components of the electron transport chain in energy generation and biosynthetic processes. These interconnected metabolic pathways collectively maintain cellular homeostasis, energy production, and macromolecule synthesis.

The pathway ID R-HSA-382551 demonstrates that cells possess multiple mechanisms to regulate the transport of small molecules across plasma membranes and between intracellular organelles. Osteopetrosis primarily arises from impaired osteoclast function, which disrupts the transport of ions and molecules required for bone resorption, resulting in abnormal bone accumulation. This defect is frequently associated with dysfunction of CA2, proton pumps, or chloride channels (CLC7), which are necessary for generating the acidic microenvironment required for bone dissolution. Consequently, reduced bone resorption leads to excessive bone density and impaired skeletal remodeling.

### Binding affinity and interaction analysis

Docking results proved that clear differences in predicted binding strength across the 11 compounds with the five protein structures. Melanin, isorenieratene, and albaflavenone consistently give the most favorable (more negative) binding scores, ranging from −7.4 to −9.6 kcal/mol, with melanin and albaflavenone displaying the single strongest poses, −9.1 and −9.6 kcal/mol, respectively. Likewise, isorenieratene and the large polyene carotenoid also score strongly across multiple receptors with a score of −7.4 to −8.9 kcal/mol, suggesting good complementarity to the binding sites (Table 6). While tiny polar molecules like geosmin, ectoine, and methylenomycin A often exhibit weaker affinities (−4.7 to −6.3 kcal/mol), except for a few better postures at specific structures, moderate binders include 7-prenylisatin, 2′-chloropentostatin, herboxidiene, and alanylclavam (−5.3 to −7.3 kcal/mol). Melanin and albaflavenone emerge as the top candidates for additional evaluation against CA2 due to the variation in scores across the five PDBs, which highlights structure-dependent binding and potentially distinct preferred binding modes. However, molecular dynamics, rescoring, and experimental validation should be conducted after these *in silico* rankings to confirm potency and selectivity. Conclusively, the docking investigation demonstrated varying binding affinities of the 11 compounds with CA 2 and antimicrobial proteins. With binding energies ranging from −7.4 to −9.6 kcal/mol, melanin, albaflavenone, and isorenieratene had the highest interactions among them, suggesting considerable potential with good stability. Further, these 3 compounds were taken forward for post-docking analysis.

**Table 6:** Binding affinity scores of 11 compounds against the protein’s CA2 and antimicrobial proteins.

S. No.	Name of the compounds	PubChem IDs	Binding affinity scores				
			CA 2	Antimicrobial proteins			
			1BCD	1MWT	4HL2	5M18	6RKS
1	Geosmin	29,746	−5.4	−6.1	−6.3	−6.1	−6
2	Ectoine	126,041	−5.1	−4.8	−6.1	−5.1	−4.7
3	Alanylclavam	194,618	−5.3	−5.3	−5.7	−5.7	−5.7
4	2’-Chloropentostatin	341,960	−6	−6.8	−6.5	−7	−7.3
5	Melanin	6,325,610	−8.4	−7.9	−7.7	−9.1	−8
6	Herboxidiene	6,438,496	−6.7	−6.6	−7.2	−6.8	−6
7	Isorenieratene	9,984,420	−7.8	−8.5	−8.9	−8.5	−8
8	Methylenomycin A	10,899,285	−5.5	−5.9	−7.9	−5.5	−5.5
9	Carotenoid	11,227,325	−7.9	−7.4	−8.5	−8	−7.5
10	Albaflavenone	25,137,938	−5.9	−6.9	−9.6	−7.1	−7.4
11	7-Prenylisatin	122,397,888	−6.2	−7	−6.4	−5.8	−7

**Figure 3:**
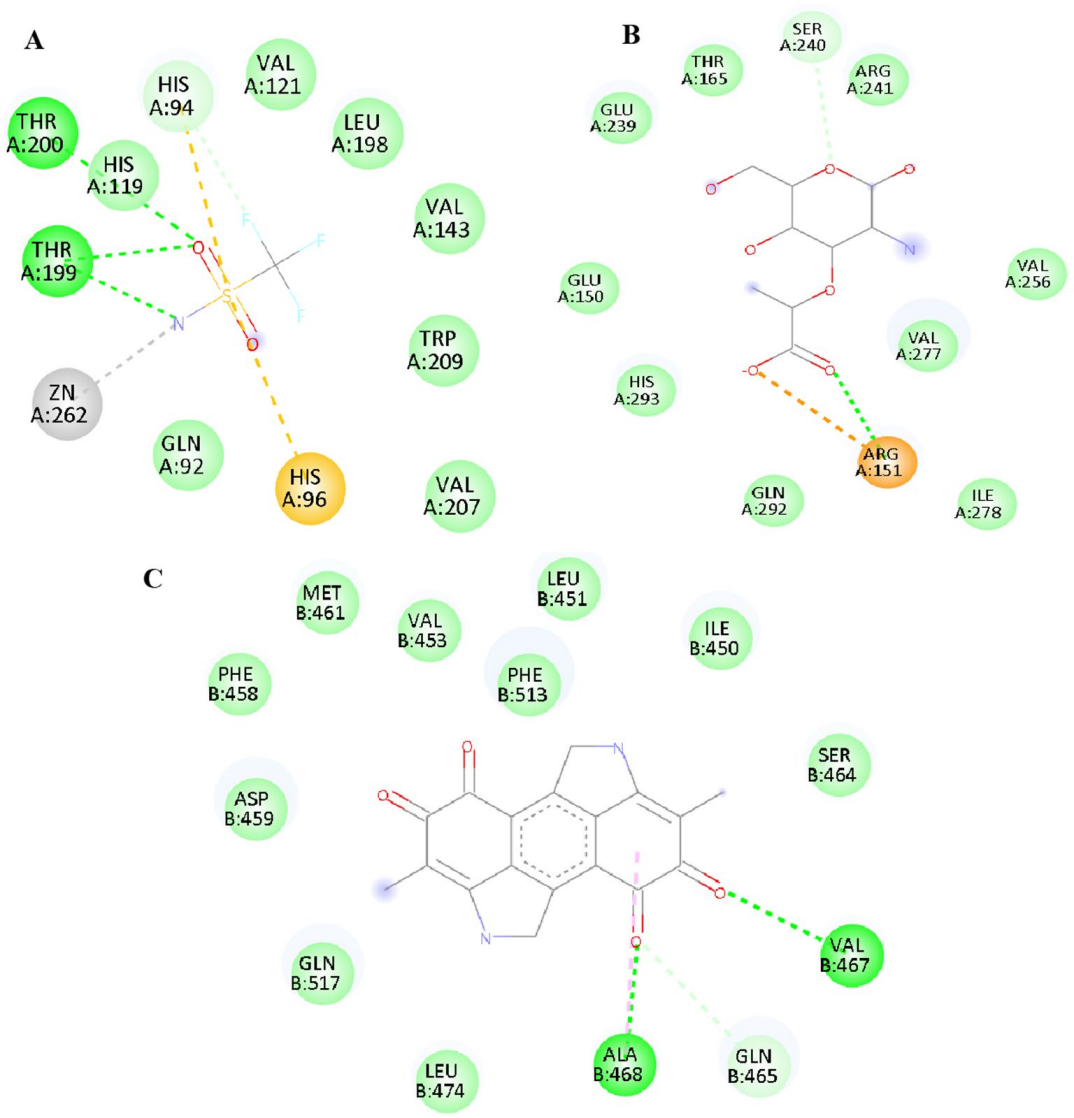
2D interaction pattern of lead compound melanin against the **(A)** CA_2_ (1BCD), and antimicrobial proteins **(B)** 5 M18, and **(C)** 6RKS.

The binding pattern of melanin with CA 2 and antimicrobial proteins is depicted in the interaction diagrams (Figure 3). Through strong hydrogen bonds, hydrophobic interactions, and Zn^2+^ coordination, melanin demonstrates persistent binding with antimicrobial proteins and CA 2. ARG and SER residues generate important hydrogen bonds in 5 M18, whereas HIS and THR residues dominate interactions in 1BCD. Major contacts for 6RKS are provided by VAL, ALA, and GLN, suggesting that melanin has a substantial inhibitory effect. Melanin’s possible inhibitory function is highlighted by its overall stable binding through a mixture of metal coordination, hydrophobic interactions, and hydrogen bonding.

**Figure 4:**
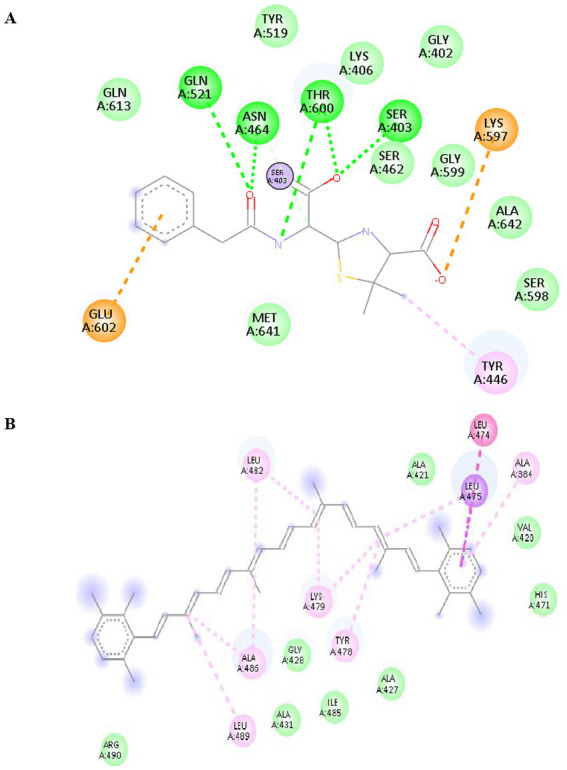
2D interaction pattern of lead compound Isorenieratene against the antimicrobial protein **(A)** 1MWT and **(B)** 6RKS.

The binding interactions between isorenieratene and antimicrobial proteins are depicted in Figure 4. In 1MWT, isorenieratene exhibits robust interaction with antimicrobial proteins via electrostatic contacts (GLU602, LYS597) and hydrogen bonds (SER403, THR600, ASN464). LYS479 supports the dominance of hydrophobic and *π*–π interactions with LEU474, LEU475, LEU486, and TYR478 in 6RKS. These interactions demonstrate the antibacterial properties and stable binding of isorenieratene.

**Figure 5:**
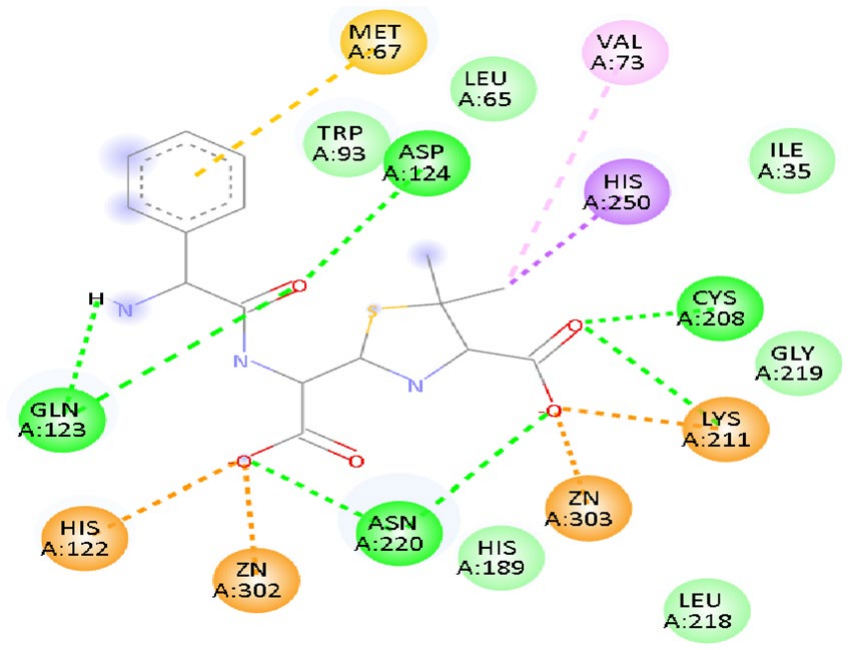
2D interaction pattern of lead compound Albaflavenone against the antimicrobial protein 4HL2.

Figure 5 reveals the binding interactions between albaflavenone and the antibacterial protein 4HL2. The compound albaflavenone increases stability in the active site by forming many hydrogen bonds with GLN123, ASP124, ASN220, and CYS208. The binding affinity is further strengthened by coordination interactions with Zn^2+^ ions (ZN302, ZN303), which are facilitated by HIS122 and HIS189. The complex is stabilized by further hydrophobic and π–alkyl interactions with MET67, VAL73, and HIS250. Albaflavenone forms robust hydrophobic, metal coordination, and hydrogen bonding interactions, suggesting that it may be a stable antibacterial inhibitor.

### Anticancer and antimicrobial activity prediction

All three lead compounds (Melanin, Isorenieratene, and Albaflavenone) have promising antineoplastic potential as shown in Table 7. Melanin has a high probability (P_a_ = 0.808) for general antineoplastic activity, with notable specificity toward solid tumors, colorectal, colon, and breast cancers; Isorenieratene has the highest probability (P_a_ = 0.881), which strongly supports its broad antineoplastic role, especially against brain, breast, and lymphoma; Albaflavenone has relatively low probabilities, with selective activity predicted for thyroid and endocrine cancers; these results collectively suggest that Melanin and Isorenieratene are powerful multi-target antineoplastic candidates, while Albaflavenone may have niche, cancer-specific effects.

**Table 7:** Antineoplastic activity of the lead compounds.

P_a_	P_i_	Activity
Melanin		
0.808	0.011	Antineoplastic
0.562	0.008	Antineoplastic (solid tumors)
0.525	0.011	Antineoplastic (colorectal cancer)
0.514	0.011	Antineoplastic (colon cancer)
0.487	0.042	Antineoplastic (non-Hodgkin’s lymphoma)
0.388	0.012	Antineoplastic (non-small cell lung cancer)
0.358	0.005	Antineoplastic (gastric cancer)
0.373	0.037	Antineoplastic (breast cancer)
0.356	0.030	Antineoplastic (lung cancer)
0.293	0.035	Antineoplastic (small cell lung cancer)
Isorenieratene		
0.881	0.005	Antineoplastic
0.610	0.013	Antineoplastic (non-Hodgkin’s lymphoma)
0.392	0.009	Antineoplastic (brain cancer)
0.408	0.035	Antineoplastic (solid tumors)
0.313	0.051	Antineoplastic (breast cancer)
0.239	0.025	Antineoplastic (glioblastoma multiforme)
0.218	0.015	Antineoplastic antibiotic
0.242	0.043	Antineoplastic (non-small cell lung cancer)
0.224	0.026	Antineoplastic (bladder cancer)
0.205	0.039	Antineoplastic (cervical cancer)
0.238	0.108	Antineoplastic (small cell lung cancer)
0.174	0.047	Antineoplastic (ovarian cancer)
0.178	0.062	Antineoplastic (renal cancer)
0.186	0.076	Antineoplastic (lung cancer)
Albaflavenone		
0.234	0.015	Antineoplastic (thyroid cancer)
0.175	0.079	Antineoplastic (endocrine cancer)
0.080	0.062	Antineoplastic antibiotic

Moreover, the screened compounds also exhibit antibacterial properties as exemplified in Table 8. A broad-spectrum action is suggested by the modest probability of melanin’s anti-*Helicobacter pylori* (P_a_ = 0.239), antimycobacterial (P_a_ = 0.274), and general antibacterial (P_a_ = 0.221) activities. Despite having a relatively moderate anti-*Helicobacter pylori* action, isorenieratene exhibits the highest antibacterial probability of P_a_ = 0.283. Although albaflavenone lacks the anticipated anti-*Helicobacter pylori* and antimycobacterial activities, it does show significant antibacterial potential with a probability of 0.266. While albaflavenone may function more selectively as a general antibacterial agent, melanin and isorenieratene showed multifaceted antimicrobial activities. Compounds with favorable drug-likeness and high Pa scores were subsequently evaluated using molecular docking to characterize their interaction strength with key antimicrobial and anticancer targets.

**Table 8:** Antibacterial activity of the lead compounds.

S. No.	Compounds	Anti-*Helicobacter pylori* activity (P_a_ value)	Antimycobacterial activity (P_a_ value)	Antibacterial activity (P_a_ value)
1	Melanin	0.239	0.274	0.221
2	Isorenieratene	0.195	–	0.283
3	Albaflavenone	–	–	0.266

### GC–MS profiling of crude extract

**Figure 6:**
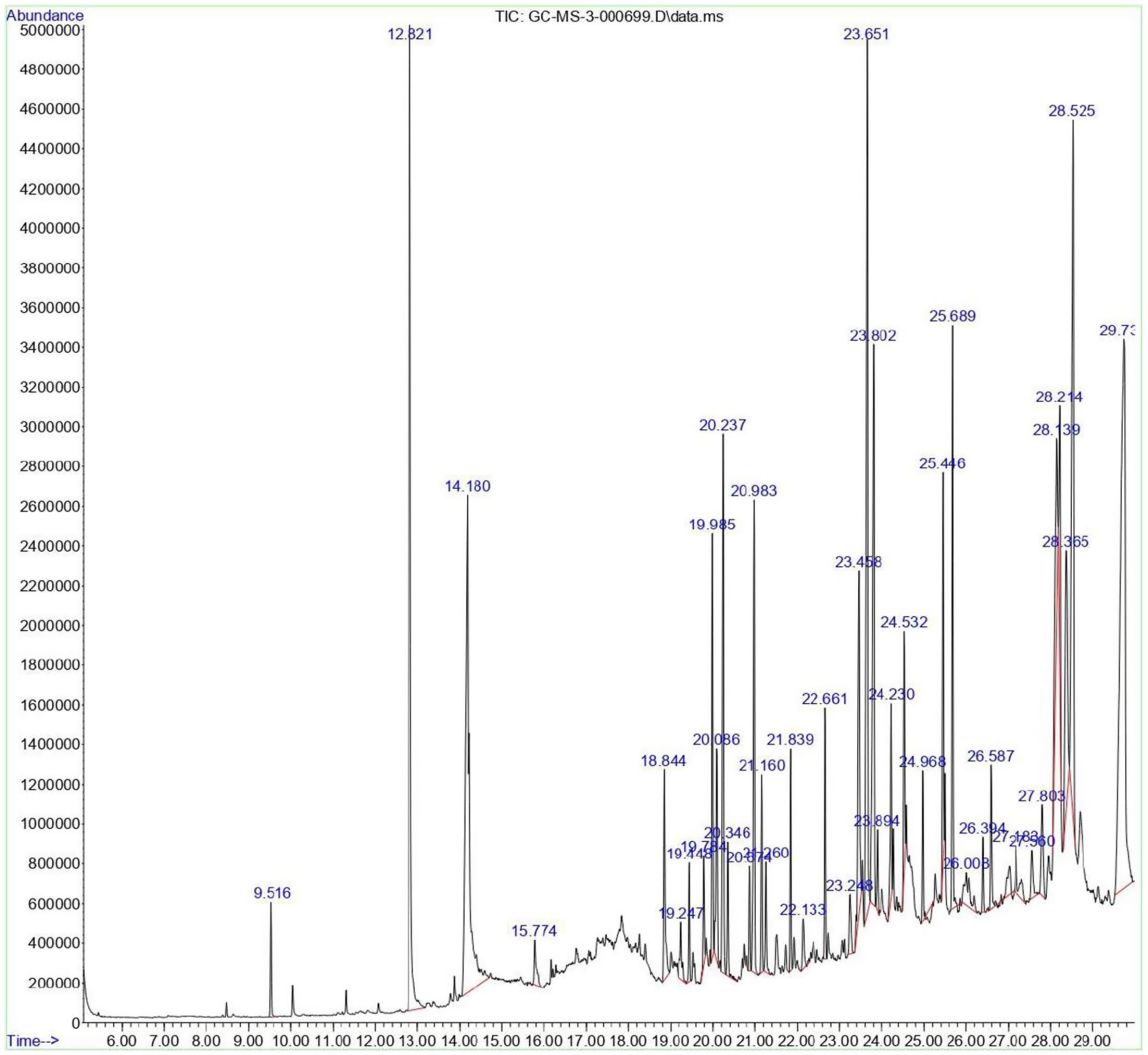
GC–MS chromatogram of the ethyl acetate extract of *Streptomyces* sp. VITGV100.

To experimentally verify whether the computationally predicted metabolites or related analogs are present in the crude extract, GC–MS profiling was performed on the culture supernatant. The ethyl acetate extract of *Streptomyces* sp. VIT100 was analyzed using GC–MS to characterize its volatile and semi-volatile constituents. The total ion chromatogram (TIC) with peak assignments is presented in Figure 6, and the complete list of detected metabolites—including retention times, molecular formulae, match factors (NIST), and relative peak areas—is provided in Supplementary Table S2. A total of 40 distinct peaks were detected. Forty compounds showed a NIST library match factor indicating reliable identification. The chemical profile consisted predominantly of alkanes (e.g., hexadecane, octadecane), phthalate derivatives, long-chain hydrocarbons, alkyl amines, methyl salicylate, and several bioactive aromatic or heterocyclic compounds. This complex metabolite signature is consistent with the diverse secondary metabolic output commonly reported for bioactive *Streptomyces* isolates. Only compounds with a NIST match factor ≥ 80% were included, ensuring reliable spectral identification. Relative metabolite abundance was expressed as normalized peak area (%), enabling comparison of compound distribution within the extract. The resulting profile reflects the chemically diverse metabolic capability characteristic of bioactive *Streptomyces* isolates.

### LC–MS-based metabolomic support for genome-mined BGCs

**Figure 7:**
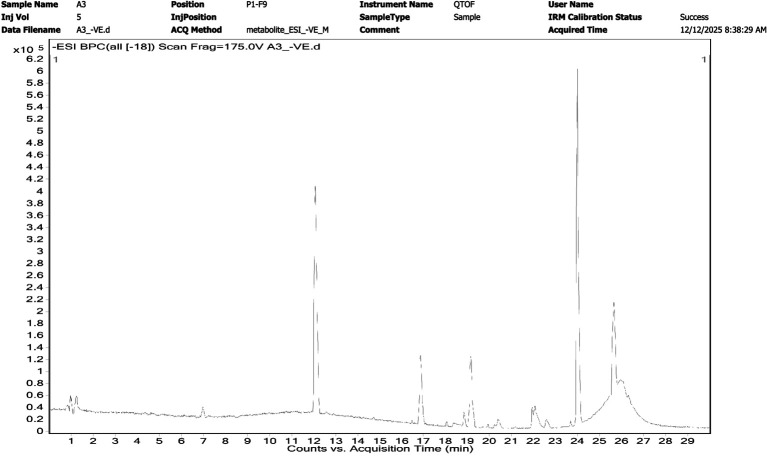
LC–MS base peak chromatogram (BPC) of the bioactive ethyl acetate extract of *Streptomyces* sp. VITGV100 analyzed in negative electrospray ionization (ESI–) mode using a QTOF mass spectrometer. The chromatogram shows multiple well-resolved peaks across the retention time range of 1–29 min, indicating the presence of chemically diverse secondary metabolites. Prominent peaks observed in the mid-to-late retention window (≈12–27 min) are consistent with hydrophobic and semi-polar metabolites, including terpenoids and polyketide-derived compounds. The observed chromatographic complexity supports genome mining predictions of multiple biosynthetic gene clusters (BGCs), related metabolites.

**Figure 8:**
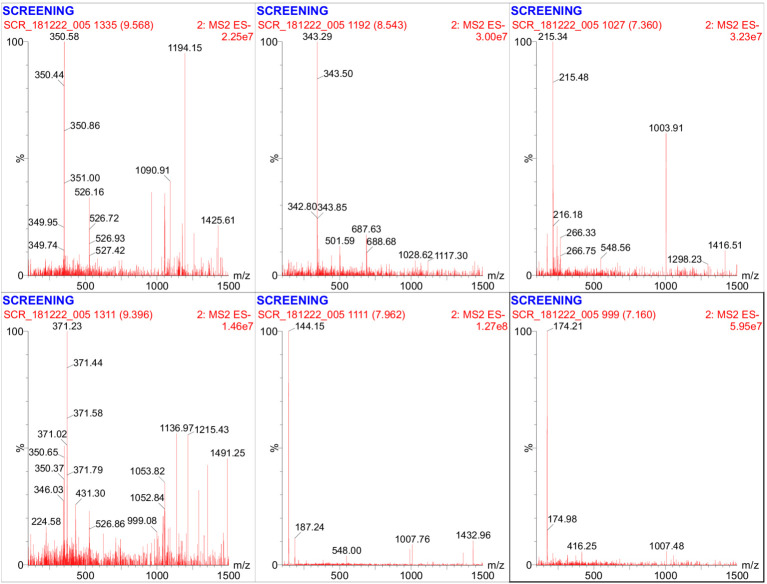
Representative LC–MS/MS fragmentation spectra of selected ions detected from the active ethyl acetate extract of *Streptomyces* sp. VITGV100 acquired in negative ESI mode. The spectra correspond to precursor ions eluting at different retention times, showing characteristic fragment ion patterns BGCS scaffolds. Several fragment profiles are consistent with metabolites biosynthesized by predicted antiSMASH BGCs, including terpene-associated clusters. These MS/MS data provide metabolomic support for genome-based predictions and highlight candidate metabolites.

LC–MS analysis of the bioactive fraction revealed a complex metabolite profile with multiple prominent peaks observed across the chromatographic run (Figure 7) and its LC–MS/MS spectra (Figure 8). The base peak chromatogram showed prominent signals primarily eluting between approximately 12 and 27 min, suggesting the presence of diverse semi-polar to non-polar secondary metabolites typical of actinomycete-derived natural products. High-resolution MS and MS/MS screening detected precursor ions spanning a broad m/z range from 144.10 to 1491.25, indicating substantial molecular diversity within the extract. Several abundant ions were observed in the mid-mass region (m/z ~ 350–550), along with higher-mass ions exceeding m/z 1,000, consistent with complex polyketide- and terpene-derived scaffolds (Figure 8). Representative precursor ions detected at m/z 350.58, 343.29, 371.23, 215.34, 174.21, and 144.15 further highlight the presence of structurally diverse metabolites. MS/MS fragmentation patterns of selected ions displayed diagnostic fragment ions characterizing the presence of metabolites associated with biosynthetic gene clusters predicted by antiSMASH. In contrast to GC–MS profiling—which primarily identified volatile, low-molecular-weight constituents such as alkanes and phthalate derivatives—LC–MS enabled the detection of non-volatile and higher-molecular-weight secondary metabolites that are more representative of cryptic biosynthetic gene cluster outputs. Together, these LC–MS findings partially bridge the gap between genome mining predictions and chemical output, supporting the functional expression of multiple BGCs in *Streptomyces* sp. VITGV100.

### Antimicrobial activity of VITGV100

**Figure 9:**
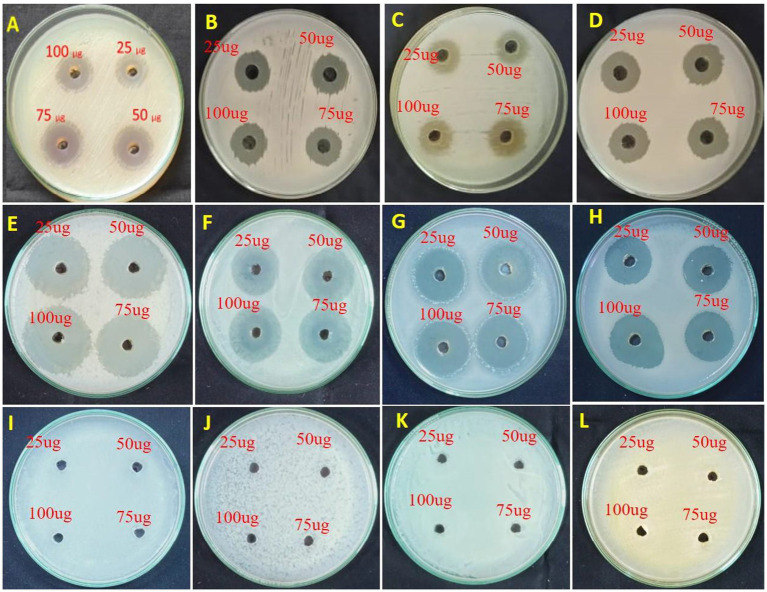
Antimicrobial activity of the crude ethyl acetate extract of *Streptomyces* sp. VITGV100 evaluated by agar well diffusion assay. **(A–D)** Antibacterial activity of the crude ethyl acetate extract of *Streptomyces* sp. VITGV100 tested at four volumes (25, 50, 75, and 100 μL per well). **(E–H)** Activity of the positive control tetracycline. **(I–L)** Negative control plates treated with DMSO, showing no inhibition. Panels correspond to the tested bacterial pathogens: *E. coli* **(A,E,I)**, *S. aureus* **(B,F,J)**, *B. subtilis* **(C,G,K)**, and *P. aeruginosa* **(D,H,L)**. Clear zones surrounding the wells indicate antibacterial activity. The crude extract exhibited dose-dependent inhibition, whereas DMSO showed no detectable antimicrobial effect.

Given the predicted bioactivities and the presence of several bioactive chemical signatures, antimicrobial assays were performed to experimentally validate the functional relevance of the genome-mining predictions. The crude ethyl acetate extract of *Streptomyces* sp. VITGV100 exhibited clear, dose-dependent antibacterial activity against all tested pathogens in the agar well diffusion assay (Table 9). At the highest tested volume (100 μL per well), the extract produced inhibition zones of 23.0 ± 0.82 mm against *E. coli*, 18.67 ± 0.94 mm against *P. aeruginosa*, 19.0 ± 0.82 mm against *B. subtilis*, and 17.67 ± 0.47 mm against *S. aureus*. The positive control, tetracycline, exhibited strong antibacterial activity across all strains, with inhibition zones ranging from 19 to 36 mm, while the negative control (DMSO) showed no inhibitory effect, confirming solvent neutrality. The antibacterial activity observed for *Streptomyces* sp. VITGV100 is comparable to that reported for bioactive *Streptomyces* extracts in previous studies, where inhibition zones typically range between 10 and 20 mm (Chevrette et al., 2019; Rammali et al., 2022; Santos-Beneit et al., 2022). These results support the potential of VITGV100 as a promising antimicrobial-producing strain (Table 9 and Figure 9).

**Table 9:** Antibacterial activity of the crude ethyl acetate extract of *Streptomyces* sp. VITGV100 evaluated by agar well diffusion assay (zone of inhibition in mm, mean ± SD, *n* = 3).

**Treatment (μL per well)**	***B. subtilis***	***S. aureus***	***P. aeruginosa***	***E. coli***
VITGV100 (25)	11.0 ± 0.82	13.0 ± 0.82	12.67 ± 0.47	17.0 ± 0.82
VITGV100 (50)	14.0 ± 0.82	14.67 ± 0.47	14.33 ± 0.47	18.33 ± 0.94
VITGV100 (75)	16.0 ± 0.82	16.67 ± 0.47	18.0 ± 0.82	19.67 ± 0.47
VITGV100 (100)	19.0 ± 0.82	17.67 ± 0.47	18.67 ± 0.94	23.0 ± 0.82
Tetracycline	30.0 ± 1.63	28.67 ± 1.24	32.0 ± 1.63	30.67 ± 1.24
DMSO (control)	0.0	0.0	0.0	0.0

Crude extract stock concentration = 1 mg/mL; volumes were applied directly into agar wells.

## Discussion

Natural products from actinomycetes, particularly *Streptomyces*, have historically provided the foundation of modern antibiotics and continue to play a pivotal role in combating antimicrobial resistance (AMR). However, the emergence of resistant pathogens necessitates the discovery of novel scaffolds and innovative strategies for drug development. In this context, our study on the tomato endophyte *Streptomyces* sp. VITGV100 demonstrates the value of integrating genome mining, *in silico* predictions, and experimental validation to systematically unlock cryptic biosynthetic potential. The high-quality draft genome of *Streptomyces* sp. VITGV100 (~7.96 Mb, 73.1% GC) is consistent with the genomic architecture typical of antibiotic-producing Streptomyces species. The assembly statistics (N50 = 110.6 kb; 129 scaffolds) fall within the expected range for Illumina-based *de novo* assemblies, indicating sufficient read depth and structural continuity. The 7,045 predicted genes and extensive KEGG/GO annotations further support the metabolic versatility of this strain. These genomic features also corroborate the taxonomic placement inferred from 16S rRNA analysis (MZ817997.1), reinforcing that VITGV100 belongs to the *Streptomyces* clade associated with prolific secondary metabolite biosynthesis. The genome-wide annotation data strengthen the biological interpretation of the biosynthetic gene clusters (BGCs) detected by antiSMASH.

The whole-genome analysis of VITGV100 revealed 35 predicted compounds associated with biosynthetic gene clusters (BGCs), spanning non-ribosomal peptide synthetases (NRPS), polyketide synthases (PKS), ribosomally synthesized and post-translationally modified peptides (RiPPs), terpenes, and melanin pathways.

This diversity underscores the strain’s metabolic versatility, consistent with prior studies showing that endophytic *Streptomyces* harbor enriched biosynthetic repertoires compared with soil isolates (Goodfellow and Fiedler, 2010). Importantly, the presence of indole-type BGCs aligns with previous reports that indole derivatives from *Streptomyces* possess strong antibacterial and anticancer potential. Such findings emphasize that plant-associated niches provide unique ecological pressures, shaping endophytes to produce structurally diverse secondary metabolites for chemical defense and interspecies competition (Patel et al., 2018; Li et al., 2025).

*In silico* pharmacokinetic analysis identified 11 compounds with favorable drug-likeness, of which melanin, isorenieratene, and albaflavenone emerged as prioritized leads. Melanin has long been recognized for its antioxidant and antimicrobial functions, but our docking studies extend its relevance by showing strong affinity toward carbonic anhydrase 2 (CA2). Carbonic anhydrases are clinically important targets in metabolic disorders, bone resorption diseases, and cancer (Supuran, 2016, 2023). The binding affinity of isorenieratene to CA2 and PPARA suggests potential roles in metabolic regulation and chemoprevention, corroborating recent reports of carotenoids acting as multifunctional bioactives (Khoo et al., 2011). Likewise, albaflavenone, a sesquiterpenoid antibiotic from *Streptomyces*, has been previously linked to antimicrobial activity (Zhao et al., 2008; Zheng et al., 2016), and our findings reinforce its therapeutic relevance with observed activity against Gram-negative and Gram-positive pathogens. Melanin is widely reported to possess antioxidant and antimicrobial activities, contributing to protection against oxidative stress and microbial pathogens. Recent pharmacological studies have also shown that melanin exhibits significant binding affinity toward carbonic anhydrase II (CA II), influencing ocular drug retention and enzymatic inhibition (Valtari et al., 2024, 2025; Urtti et al., 2025). Consistent with these findings, our docking analysis revealed a strong predicted interaction between melanin and CA2, suggesting a potential functional association that warrants further experimental validation.

The antibacterial activity exhibited by *Streptomyces* sp. VITGV100 aligns with the inhibitory ranges commonly reported for metabolite-rich Streptomyces extracts (Chevrette et al., 2019; Rammali et al., 2022; Rai et al., 2025). The stronger inhibition against Gram-negative *E. coli* and *P. aeruginosa* corresponds with the presence of predicted NRPS, PKS, and terpenoid BGCs in the genome, which are frequently responsible for broad-spectrum antimicrobial scaffolds. Furthermore, the observed zones of inhibition, although lower than pure tetracycline, exceed many crude extract activities reported for natural Streptomyces isolates, indicating potent metabolite production. The lack of activity in the DMSO control confirms that the effects arise solely from bioactive compounds of VITGV100. While MIC and MBC determinations were not included in this preliminary screening, such assays are part of ongoing work and will further quantify potency, following standard approaches used in Streptomyces-based drug discovery pipelines. The observed susceptibility of Gram-negative pathogens, particularly *P. aeruginosa*, is noteworthy, given their intrinsic resistance to many natural products due to the impermeability of the outer membrane (Blair et al., 2015). This suggests that metabolites such as isorenieratene may exert membrane-penetrating or destabilizing effects, consistent with their hydrophobic nature.

Beyond antimicrobial applications, the predicted antineoplastic activities of melanin and isorenieratene open new avenues for multifunctional drug discovery. Natural compounds with dual antimicrobial and anticancer activity are especially valuable for immunocompromised patients, where co-infections and malignancies often intersect (Chifiriuc et al., 2022). The dual targeting potential also aligns with the current shift toward developing multi-indication therapeutics from natural scaffolds (Newman and Cragg, 2007, 2020).

*In silico* analyses of *Streptomyces* sp. VITGV100 revealed multiple biosynthetic gene clusters (BGCs), including NRPS, PKS, terpenoid, siderophore, and RiPP-associated clusters, which are commonly linked to antibacterial metabolites in *Streptomyces* spp. (Rehan et al., 2023; Kumar et al., 2025). Several of these predicted clusters—particularly NRPS and type-I PKS pathways—are known to encode scaffolds such as peptide antibiotics, polyketide macrolides, and aromatic polyketides, which frequently contribute to activity against Gram-positive and Gram-negative pathogens (Meesil et al., 2023).

Consistently, GC–MS analysis of the ethyl acetate extract revealed long-chain hydrocarbons, phthalate derivatives, alkyl amines, methyl salicylate, and heterocyclic aromatic compounds, many of which have been reported as secondary metabolites produced by PKS- and NRPS-associated BGCs in *Streptomyces* (Williams et al., 2022). Several aromatic and heterocyclic constituents identified—including methyl salicylate, 2,5-piperazinedione derivatives, and alkylated carbazoles—are known to possess antimicrobial properties and are characteristic products of actinomycete secondary metabolism. Moreover, molecular docking suggested that several predicted metabolites exhibit strong binding affinities toward clinically relevant antimicrobial targets such as PBP2a, *β*-lactamases, and DNA gyrase, which aligns mechanistically with the observed inhibition of *S. aureus* and *E. coli*. This pattern is in line with previous reports showing that genome-predicted NRPS/PKS metabolites often correlate with phenotypic antibacterial activity in *Streptomyces* isolates (Hannigan et al., 2019). While targeted purification and LC–MS/MS structural confirmation were beyond the scope of this initial study, the convergence of genome-mining predictions, docking outputs, GC–MS profiles, and antimicrobial assays provides a coherent systems-level interpretation. Together, these data support the conclusion that *Streptomyces* sp. VITGV100 actively synthesizes bioactive metabolites that are consistent with the predicted BGC repertoire and exhibit measurable antibacterial effects. The integration of LC–MS analysis significantly strengthens the connection between genome mining and experimental validation in this study. While GC–MS profiling provided an overview of volatile constituents present in the crude extract, it was inherently limited in its ability to detect complex secondary metabolites encoded by large biosynthetic gene clusters, such as terpenes and polyketides. The LC–MS/MS data revealed multiple ions and fragmentation patterns consistent with the presence of products associated with predicted BGCs and its biosynthesis in the crude extract. Although the present LC–MS analysis offers strong metabolomic support for the functional expression of genome-mined BGCs. This integrative genome–metabolome approach highlights *Streptomyces* sp. VITGV100 as a promising source of antimicrobial secondary metabolites and lays the foundation for future targeted isolation and structural elucidation studies.

While these findings are promising, certain limitations should be acknowledged. The antimicrobial assays relied on crude extracts, which may underestimate the true potency of individual metabolites. Furthermore, *in vivo* pharmacological validation was not performed, and thus, the bioavailability and safety of the identified compounds remain to be established. Nevertheless, the combined computational and experimental approach strengthens confidence in the bioactivity predictions, providing a strong foundation for downstream validation.

Looking forward, metabolic engineering offers significant opportunities to enhance the yields and chemical diversity of VITGV100-derived metabolites. Strategies such as promoter engineering, CRISPR–Cas9 mediated BGC refactoring, and heterologous expression in optimized *Streptomyces* chassis can activate silent clusters and improve titers (Mungan et al., 2020). Such approaches are increasingly recognized as essential for translating genomic potential into tangible drug leads. Moreover, the development of VITGV100 as a microbial cell factory aligns with global sustainability goals, reducing reliance on synthetic chemical routes that are energy-intensive and environmentally harmful. Because SwissTargetPrediction is optimized for *Homo sapiens* proteins, predicted targets were biased toward human signaling pathways rather than bacterial systems. Although docking analyses included essential bacterial enzymes, the lack of bacterial-specific target-prediction tools represents a methodological limitation. Future work will incorporate proteome-wide bacterial target prioritization platforms once the structures of key metabolites are experimentally validated.

Our study expands the repertoire of endophytic *Streptomyces* as renewable platforms for drug discovery. We note that all cheminformatics analyses performed here—SwissADME, SwissTargetPrediction, PASS, and DisGeNET—are based on the chemical structures predicted for high-similarity BGCs. These analyses do not evaluate the BGCs or their genes directly. Accordingly, all results reflect compound-level predictions, not cluster-level biological functions. By linking genome mining with functional validation, we not only identified novel antimicrobial and anticancer leads but also established a replicable pipeline for bioprospecting actinomycetes. This work supports the broader paradigm of leveraging synthetic biology and metabolic engineering to build sustainable microbial cell factories, thereby advancing the dual goals of combating AMR and fostering a circular bioeconomy.

Genome mining using antiSMASH revealed multiple biosynthetic gene clusters (BGCs) in *Streptomyces* sp. VITGV100, including those putatively associated with melanin biosynthesis, terpene pathways (albaflavenone and isorenieratene), and polyketide/nonribosomal peptide synthesis. While antimicrobial assays were conducted using a crude ethyl acetate extract, the observed dose-dependent antibacterial activity is likely attributable to the collective and potentially synergistic action of multiple secondary metabolites encoded by these BGCs.

GC–MS profiling primarily detected volatile and semi-volatile compounds; however, it is well recognized that pigments and higher-molecular-weight terpenoids are not always efficiently captured by GC–MS, particularly without chemical derivatization. To overcome this limitation, LC–MS analysis was performed, revealing multiple hydrophobic and semi-polar metabolites eluting in the mid-to-late retention range, consistent with terpene- and pigment-derived compounds. These LC–MS features provide metabolomic support for the presence of secondary metabolites corresponding to predicted terpene and pigment BGCs scaffolds.

Importantly, existing literature supports the antibacterial relevance of these compounds. Streptomyces-derived melanin pigments have been reported to exhibit antibacterial activity against both Gram-positive and Gram-negative pathogens, in addition to antioxidant and cytoprotective properties (Vasanthabharathi et al., 2011; El-Naggar and El-Ewasy, 2017). Similarly, albaflavenone, a sesquiterpene antibiotic biosynthesized via the epi-isozizaene pathway in *Streptomyces*, has been mechanistically characterized and shown to possess antibacterial and cytotoxic properties (Lin and Cane, 2009; Zheng et al., 2016). Aromatic carotenoids such as isorenieratene, while primarily associated with membrane stabilization and oxidative stress protection, may indirectly contribute to antimicrobial fitness and stress tolerance, enhancing overall bioactivity in crude extracts.

Taken together, the convergence of genome mining predictions, LC–MS metabolomic profiles, and observed antibacterial activity supports the hypothesis that *Streptomyces* sp. VITGV100 harbors a functionally active secondary metabolite arsenal. Nevertheless, we acknowledge that unambiguous compound–activity correlations require purification, structural elucidation, and bioassay-guided fractionation, which are planned for future investigations. The present study therefore provides a robust genome–metabolome–bioactivity framework, positioning VITGV100 as a promising source of antibacterial natural products.

## Conclusion

This study highlights the untapped biosynthetic potential of the endophytic strain *Streptomyces* sp. VITGV100 as a sustainable microbial platform for natural product discovery. By integrating genome mining, cheminformatics, LC–MS–based metabolomic profiling, and *in vitro* antibacterial assays, we prioritized several computational candidate metabolites, including melanin-, isorenieratene-, and albaflavenone-associated biosynthetic pathways, as promising contributors to the observed bioactivity. Importantly, these compounds are identified here as in silico–prioritized candidates rather than experimentally confirmed lead molecules. While the crude extract of VITGV100 exhibited clear dose-dependent antibacterial activity, definitive attribution of this activity to individual metabolites will require future purification, structural elucidation, and bioassay-guided fractionation. Nonetheless, the convergence of genome mining predictions, metabolomic signatures, molecular docking analyses, and antibacterial phenotypes supports the functional relevance of the predicted biosynthetic gene clusters. Beyond compound discovery, this work demonstrates the power of genome-to-function pipelines in accelerating the prioritization of cryptic biosynthetic pathways in *Streptomyces*. Such integrated strategies provide a rational framework for sustainable natural product discovery and lay the groundwork for future metabolic engineering and synthetic biology approaches to enhance metabolite production and diversification.

## Data Availability

The datasets presented in this study can be found in online repositories. The names of the repository/repositories and accession number(s) can be found at: https://www.ncbi.nlm.nih.gov/, SRA accession number SRS9635924.

## Footnotes

1
https://www.disgenet.org/

2
http://www.rcsb.org/
