# Supplementary material for: Genome mining strategies to unlock the metabolic potential of Streptomyces sp. VITGV100 (MCC 4961) for next-generation antimicrobial discovery
Source: Front Microbiol. 2026 May 14;17:1713836. doi: 10.3389/fmicb.2026.1713836 (PMC13216219; doi:10.3389/fmicb.2026.1713836)
Supplement: Supplementary file 1 [file Data_Sheet_1.pdf]

Genome Mining Strategies to Unlock the Metabolic Potential of *Streptomyces* sp. VITGV100 for Next-Generation Antimicrobial Discovery

Supplementary File

Veilumuthu Pattapulavar[1], Saranayadevi Subburaj[2], Sathiyabama Ramanujam[3], Riyanka Shill[1], Priyanka Velmurugan[4], Tapas Ghatak[4], Sanjivkumar Muthusamy[5] Antony V Samrot[6] and John Godwin Christopher[1]

[1]Department of Biomedical Sciences, School of BioSciences and Technology, Vellore Institute of Technology, Vellore, India
[2]Department of Biotechnology (FoE), Karpagam Academy of Higher Education, Coimbatore
[3]Department of Science and Humanities, Karpagam Academy of Higher Education, Coimbatore, Tamil Nadu, India
[4]School of Advanced Sciences, Vellore Institute of Technology, Vellore, Tamil Nadu, India
[5]Department of Microbiology, K.R. College of Arts & Science, K.R. Nagar, Kovilpatti, Tamil Nadu, India
[6]Department of Microbiology, Faculty of Medicine, Manipal University College Malaysia, Melaka, Malaysia

Table S1. Docking grid parameters and validation metrics

Docking grid centers, box sizes, exhaustiveness and re-docking RMSD values for each target protein (AutoDock Vina via PyRx v0.8).

#	Protein target (common name)	PDB ID	Grid center (X, Y, Z) (Å)	Grid size (X × Y × Z) (Å)	Exhaustiveness	Native ligand RMSD (Å)	Validation (Pass/Borderline/Fail)
1	Carbonic anhydrase II (CA2)	1CA2	12.4, −3.2, 18.7	22 × 22 × 22	8	1.42	Pass
2	DNA gyrase subunit B (GyrB)	4DUH / 6RKS*	−15.3, 24.1, 32.8	28 × 28 × 28	8	1.75	Pass
3	Dihydrofolate reductase (DHFR)	4LAE	8.6, −12.7, 5.3	24 × 24 × 24	8	1.38	Pass
4	MurB reductase (MurB)	1MBR	45.2, −8.3, 14.8	30 × 30 × 30	8	2.21	Borderline
5	Class A β-lactamase (TEM-1)	1BTL / 5M18*	−2.8, 6.4, 19.9	22 × 22 × 22	8	1.63	Pass
6	PARP1	4ZZZ	10.1, 14.2, −8.5	28 × 28 × 28	8	1.57	Pass
7	PPAR-α	3VI8	−21.2, 18.3, 3.4	26 × 26 × 26	8	1.95	Pass
8	FabH (β-ketoacyl-ACP synthase III)	1HNJ	−11.6, 9.7, 24.1	24 × 24 × 24	8	1.31	Pass
9	Topoisomerase IV (ParC/ParE)	1S16	14.3, −23.8, 11.2	30 × 30 × 30	8	1.84	Pass

10	MurA (UDP-N-acetylglucosamine enolpyruvyl transferase)	1UA1	−28.3, 15.1, 5.8	26 × 26 × 26	8	2.12	Borderline
11	PBP2a (penicillin-binding protein 2a)	1MWT / 1VQQ*	6.7, 11.5, −16.2	28 × 28 × 28	8	1.69	Pass

* Where multiple PDB entries exist for a target family (e.g., class A β-lactamases, gyrase), we used the low-resolution structure chosen at the time of docking; exact PDB file used for each ligand is listed in the Supplementary Data.

Table S2: GC-MS identified compounds for *Streptomyces* sp. VITGv100

S.No	Chemical Compound	Retention Time (min)	Molecular weight (g/mol)	Molecular Formula	Area %
1	2,3-Butanediol	5.354	90.12	$C_4H_{10}O_2$	1.87
2	Fumaric acid, 3,4- dimethoxyphenyl	23.838	-	-	1.30
3	Decane, 3,7- dimethyl-	8.345	170.33	$C_{12}H_{26}$	1.34
4	Isopropyl butyrate	9.295	130.18	$C_7H_{14}O_2$	3.15
5	Heptadecane, 8- methyl-	12.059	254.5	$C_{18}H_{38}$	1.45
6	2-Bromo dodecane	12.820	249.23	$C_{12}H_{25}Br$	0.96
7	Methyl salicylate	13.388	152.15	$C_8H_8O_3$	1.38
8	Hexadecane	14.970	226.44	$C_{16}H_{34}$	0.79
9	Octadecane, 1- iodo-	15.618	380.4	$C_{18}H_{37}I$	1.56
10	Octadecane, 1- iodo-	16.008	296.6	$C_{18}H_{37}I$	1.56
11	Octadecane	18.055	254.5	$C_{18}H_{38}$	2.02
12	Diethyl Phthalate	19.149	222.24	$C_{12}H_{14}O_4$	3.05
13	Pentacosane	20.248	352.7	$C_{25}H_{52}$	1.36
14	Nonacosane, 2- methyl-	22.292	422.8	$C_{30}H_{62}$	1.55
15	2,5Piperazinedione, 3,6-bis(2	23.358	226.32	$C_{12}H_{22}N_2O_2$	1.98

	methylpropyl)-				
16	2-Tetradecanol	5.102	214.39	$C_{14}H_{30}O$	0.13
17	trans-2,3- Epoxydecane	9.062	156.26	$C_{10}H_{20}O$	0.07
18	Propanoic acid, 3- hydroxy-, hydr	11.613	-	-	0.15
19	Tetradecane, 2,6,10-trimethyl-	12.960	240.5	$C_{17}H_{36}$	0.41
20	Octadecane	13.086	254.5	$C_{18}H_{38}$	0.42
21	Cycloheptasiloxa ne, tetradecamethyl	13.695	519.07	$C_{14}H_{42}O_7Si_7$	0.36
22	Tricosane	14.114	324.6	$C_{23}H_{48}$	0.34
23	2-Chloro-11H- pyrido[3',2'- 4,5]py	14.712	-	-	0.21
24	Octadecane	15.091	254.5	$C_{18}H_{38}$	0.52
25	Nonadecane, 3- methyl-	15.746	282.5	$C_{20}H_{42}$	0.38
26	9H-carbazole-3,6- diamine, N3,N3	16.511	253.34	$C_{16}H_{19}N_3$	0.33
27	Octadecane, 1- iodo-	16.680	380.4	$C_{18}H_{37}I$	0.68
28	7- Oxodehydroabieti c acid, methyl	16.948	328.4	$C_{21}H_{28}O_3$	0.20
29	7-Oxodehydroabieti c acid, methyl	17.114	328.4	$C_{21}H_{28}O_3$	0.52
30	Octadecane	17.495	254.5	$C_{18}H_{38}$	1.06
31	Nonadecane, 3- methyl-	17.902	282.5	$C_{20}H_{42}$	0.41
32	Octadecane, 1- iodo-	18.626	380.4	$C_{18}H_{37}I$	0.59
33	Orotyl demicarbazide	19.498	213.15	$C_6H_7N_5O_4$	0.07
34	Hexacosane	19.752	366.7	$C_{26}H_{54}$	0.64
35	1-Tetradecanamin e	20.132	213.4	$C_{14}H_{31}N$	0.12
36	19-Norbrassicaster- 3-one, 7,8,9	21.157	378.6	$C_{27}H_{38}O$	0.40
37	Phthalic acid, 2- ethylhexyl pent	21.771	-	-	0.33
38	3-((1-[5-Benzyl- 1-(4-fluoropheny	23.133	-	-	0.46
39	Propanoic acid, 2- (aminooxy)-	6.324	105.09	$C_3H_7NO_3$	1.29
40	5H- [1]Benzopyrano[3,4-c]pyridin-	24.149	346.4	$C_{21}H_{18}N_2O_3$	0.39
